# Astragaloside IV Attenuates the Myocardial Injury Caused by Adriamycin by Inhibiting Autophagy

**DOI:** 10.3389/fphar.2021.669782

**Published:** 2021-05-24

**Authors:** Li-Fei Luo, Lu-Yun Qin, Jian-Xin Wang, Peng Guan, Na Wang, En-Sheng Ji

**Affiliations:** ^1^ Department of Physiology, School of Basic Medical Sciences, Hebei University of Chinese Medicine, Shijiazhuang, China; ^2^ Department of Physiology, College of Life Sciences, Hebei Normal University, Shijiazhuang, China

**Keywords:** heart, astragaloside IV, adriamycin, apoptosis, autophagy, oxidative stress

## Abstract

Astragaloside IV (ASIV) is the main active component of *Astragalus*, and can ameliorate cardiomyocyte hypertrophy, apoptosis and fibrosis. In this experiment, we studied how ASIV reduces the cardiotoxicity caused by adriamycin and protects the heart. To this end, rats were randomly divided into the control, ADR, ADR + ASIV and ASIV groups (*n* = 6). Echocardiography was used to observe cardiac function, HE staining was used to observe myocardial injury, TUNEL staining was used to observe myocardial cell apoptosis, and immunofluorescence and Western blotting was used to observe relevant proteins expression. Experiments have shown that adriamycin can damage heart function in rats, and increase the cell apoptosis index, autophagy level and oxidative stress level. Further results showed that ADR can inhibit the PI3K/Akt pathway. ASIV treatment can significantly improve the cardiac function of rats treated with ADR and regulate autophagy, oxidative stress and apoptosis. Our findings indicate that ASIV may reduce the heart damage caused by adriamycin by activating the PI3K/Akt pathway.

## Introduction

Adriamycin (ADR) is a powerful antineoplastic agent (Liu et al., 2020) that is widely used to treat high-grade osteosarcoma (Anninga et al., 2011) in the clinic. However, cumulative doses of adriamycin can cause severe heart damage (Navarro-Hortal et al., 2020). Although adriamycin is a first-line cancer treatment drug, its cardiotoxicity can lead to cardiomyopathy and eventually to heart failure, which substantially limits its clinical application. Previous studies have shown that induction of an abnormal increase in free radical productions is the main mechanism by which adriamycin causes myocardial damage (Sun et al., 2014; Kalyanaraman, 2020). However, with the gradual increase in the number of studies showing that adriamycin causes myocardial damage through oxidative stress, it was found that inhibiting oxidative stress alone cannot prevent heart failure (Sun et al., 2014; Khadka et al., 2018; Kalyanaraman, 2020). Therefore, the cardiotoxicity caused by adriamycin through other mechanisms is also worth studying.

Astragaloside IV (ASIV) is derived from Astragalus and is one of its main active ingredients (Zhou et al., 2020). ASIV has many pharmacological effects, such as antioxidant, anti-inflammatory, anti-fibrosis and immune regulatory effect (Wang et al., 2019). It has been suggested that ASIV has ameliorating effects on cardiac myocyte hypertrophy (Nie et al., 2019) and apoptosis (Zheng et al., 2019).

Autophagy is a process of cellular self-degradation (Yang et al., 2020), and it plays essential roles in the renewal of intracellular substances and the maintenance of intracellular homeostasis (Yusipov et al., 2020). Increased autophagy can reduce the effects of various pathological factors on cardiomyocytes. However, excessive autophagy mistakenly degrades important cellular components and exacerbates damage (Yang et al., 2020). Autophagic dysfunction plays an important role in the cardiotoxicity caused by adriamycin (Xu et al., 2020). Studies have shown that in the cardiomyopathy induced by adriamycin, inhibiting autophagy has a beneficial effect (Ma et al., 2017). The PI3K/Akt/mTOR signaling pathway may play an important role in this effect (Yu et al., 2020).

Nuclear factor erythroid 2-related factor 2 (Nrf2) is a transcription factor that not only regulates the phase II detoxification response (Silva-Palacios et al., 2016), but also regulates dominates redox homeostasis and cellular antioxidant defense (Peng et al., 2020). Kelch-like ECH-associated protein 1 (Keap1) is a protein responsible for the cytoplasmic isolation of Nrf2 under physiological conditions (Kourakis et al., 2020). In general, Keap1 promotes the ubiquitination and degradation of Nrf2. After stimulation, Nrf2-dependent cellular defense mechanisms are activated, and Nrf2 separates from Keap1 and enters the nucleus (Zheng et al., 2020). Among the downstream genes of Nrf2, p62, which is encoded by the SQSTM1 gene, is the specific autophagy receptor for Keap1 (Deng et al., 2020). P62 binds to Keap1 and intercalates with other ubiquitinated protein aggregates for degradation, thereby releasing and activating Nrf2. In the nucleus, Nrf2 promotes p62 gene expression, which causes Nrf2 to continue to be activated (Luo et al., 2020). It can be deduced that Nrf2, Keap1 and p62 form a circular pathway in the body and together play a role in maintaining homeostasis during oxidative stress (Zhang et al., 2020b).

In this experiment, we investigated whether ASIV could reduce the cardiac damage caused by adriamycin by inhibiting autophagy and oxidative stress.

## Materials and Methods

### Animals

The use of animals and the experimental protocols were approved by the Animal Care and Use Committee of Hebei University of Chinese Medicine. A total of 24 SD male rats weighing 200–210 g were obtained from Vital River Laboratory Animal Technology Co., Ltd. and were randomly divided into the control, ADR, ASIV + ADR and ASIV groups (*n* = 6). As indicated, astragaloside IV was given by intragastric administration (1.0 mg/kg, daily), and adriamycin was intraperitoneally injected (4 mg/kg/week) for 5 weeks, while the other two groups were administered the same volume of solvent.

### Cell Culture

H9C2 cells were purchased from the Institute of Cell Research, Chinese Academy of Sciences (CTCC, Shanghai, China). The cells were maintained in DMEM with 10% FBS and 1% penicillin-streptomycin. The medium was changed every 1–2 days. The cells were subcultured at 80–90% confluence for the experiments. The cells were serum-starved overnight and randomly divided into six groups: the control group, DMSO group, DMSO + ADR group, DMSO + ASIV + ADR group, DMSO + ASIV + PARP group, and DMSOASIV + LY294002 group.

### Cell Viability Assay

The CCK-8 cell counting kit was used to detect the viability of H9C2 cells after treatment. H9C2 cells (100 µL) were seeded in a 96-well plate at a density of 1–2 ×10^4^ cells/mL, and the cells were treated after they adhered to the well. After the cell treatment was complete, the medium was replaced with 100 µL medium containing 10% CCK-8. The cells were cultured in the dark for 2 h, and the absorbance was measured at 450 nm. The ADR and ASIV treatment time was 24 h, and the rapamycin and LY294002 treatment time was 2 h. The final concentrations were as follows: 5 µM adriamycin, 100 µM astragaloside IV, 100 nM rapamycin, and 20 µM LY294002.

### Nuclear Extraction

The nuclei were extracted using a nuclear extraction kit (NT-032, Invent Biotechnologies, United States). In short, the tissue was homogenized and centrifuged at 14,000 × g for 5 min, and the pellet was resuspended, and centrifuged at 500 × g for 2 min. The pellet centrifuged again at 2000 × g for 2 min. The pellet at the bottom of the centrifuge tube was the nuclear extract.

### Echocardiography

Echocardiography was performed after the final injection. Isoflurane (2.5%) inhalation was used for anesthesia, and the rats were arranged in the supine position. The left ventricular ejection fraction (EF), fraction shortening (FS), and systolic and diastolic thickness of the left ventricular anterior wall were evaluated with the M-mode echocardiogram in the parasternal short-axis view. The operators are unaware of the animal grouping.

### TUNEL Assay

To evaluate the extent of DNA degradation, cells were incubated with TUNEL reaction solution on a glass slide at 37°C for 1 h. After incubation, the cells were counterstained with DAPI (2 mg/ml, Solarbio, Beijing, China). TUNEL positive cells were observed and quantified using a microscope (Leica Microsystems GmbH). The rate of apoptosis is presented as the ratio of TUNEL positive nuclei to total DAPI-stained nuclei.

### Oxidative Stress Assays

This experiment was performed with a kit produced by Nanjing Jiancheng Bioengineering Research Institute. Superoxide dismutase (SOD), glutathione peroxidase (GSH-Px), malondialdehyde (MDA) and catalase (CAT) were extracted from rat heart homogenates using a kit. Then, a double cinnamic acid protein assay kit was used to detect the protein concentration (CWBio, Beijing, China). Finally, the absorbances different wavelengths were detected with a microplate reader. The wavelengths were as follows: MDA and SOD: 550 nm; CAT: 405 nm; and GSH-Px: 412 nm.

### Histological Examination

First, the separated left ventricle was washed with PBS. The heart was fixed (72 h) with paraformaldehyde (4%), embedded in paraffin, and sectioned (5 µm). Hematoxylin-eosin staining was used for microscopic analysis (400-x magnification) (Leica Microsystems GmbH).

### Western Blot Analysis

The rat myocardial tissue was homogenized, and then, a protease inhibitor cocktail (R and D Systems Inc., Minneapolis, Minnesota, United States) and RIPA lysis buffer (Thermo Fisher Scientific, United States) were added to extract the proteins. The extracted proteins were separated by SDS-PAGE and transferred to PVDF membranes (Millipore Co, Bedford, MA). The following primary antibodies were used to detect the target proteins: anti-*p*-mTOR (1:1,500) from Abcam; anti-BCL2 (1:1,500) from Immunoway; anti-PARP (1:1,500) from GeneTex; anti-mTOR (1:1,500), anti-p62 (1:1,500), anti-actin (1:1,500), anti-tubulin (1:1,500), anti-beclin1 (1:1,500), anti-SOD2 (1:1,500), anti-Cyt-C (1:1,500), and anti-HO-1 (1:1,500), anti-Bax (1:1,500) from Servicebio; anti-LC3II(1:3,500) from Sigma; anti-PI3K (1:1,500), anti-*p*-Akt (1:1,500), anti-Nrf2(1:1,500), and anti-Akt (1:1,500) from Proteintech; anti-caspase-3 from cell Signaling Technology; anti-Keap1 (1:800) from Absin; and anti-Lamin B1 (1:1,000) from HuaBio. The internal control was tubulin. After blocking, the membranes were probed for tubulin, Bax, HO-1, and *p*-Akt and incubated with horseradish peroxidase-conjugated goat anti-mouse IgG antibodies (1:5,000, Servicebio, Wuhan, China). Other membranes were incubated with horseradish peroxidase-conjugated goat anti-rabbit IgG antibodies at a 1:3,000 dilution (Servicebio, Wuhan, China). The signal was visualized by enhanced chemiluminescence detection (Vilber Fusion FX5 Spectra, Paris, France). The protein expression graph shows the fold change relative to the control group.

### Immunofluorescence

First, the separated left ventricle was washed with PBS. The heart was fixed in paraformaldehyde (4%) for 72 h, embedded in paraffin and sectioning (5 µm). The paraffin sections of the heart were deparaffinized, hydrated, and permeabilized for immunofluorescence. Rabbit polyclonal anti-LC3II (1:300), anti-*p*-Akt (1:300), anti-Nrf2 (1:300) and anti-PI3K (1:300) antibodies were first added and incubated to detect the contents of these proteins in the heart. FITC-labeled anti-rabbit IgG antibodies were used as the second antibodies for detection (Zhongshan Golden Bridge Biotechnology, Beijing, China). A fluorescence microscope (Leica Microsystems GmbH, Wetzlar, Germany) was used to observe the sections after staining with 4′,6-dimethylene-2-phenylindole (DAPI). ImageJ software was used to quantify the results.

### Statistical Analysis

The analysis software used SPSS 19.0 (IBM, New York, NY). The data are expressed as the mean ± SEM. The differences between the four groups were analyzed using one-way analysis of variance (ANOVA) followed by a Tukey test. *p* < 0.05 is indicated statistically significant differences.

## Results

### ASIV Alleviates ADR -Induced Myocardial Injury

**FIGURE 1:**
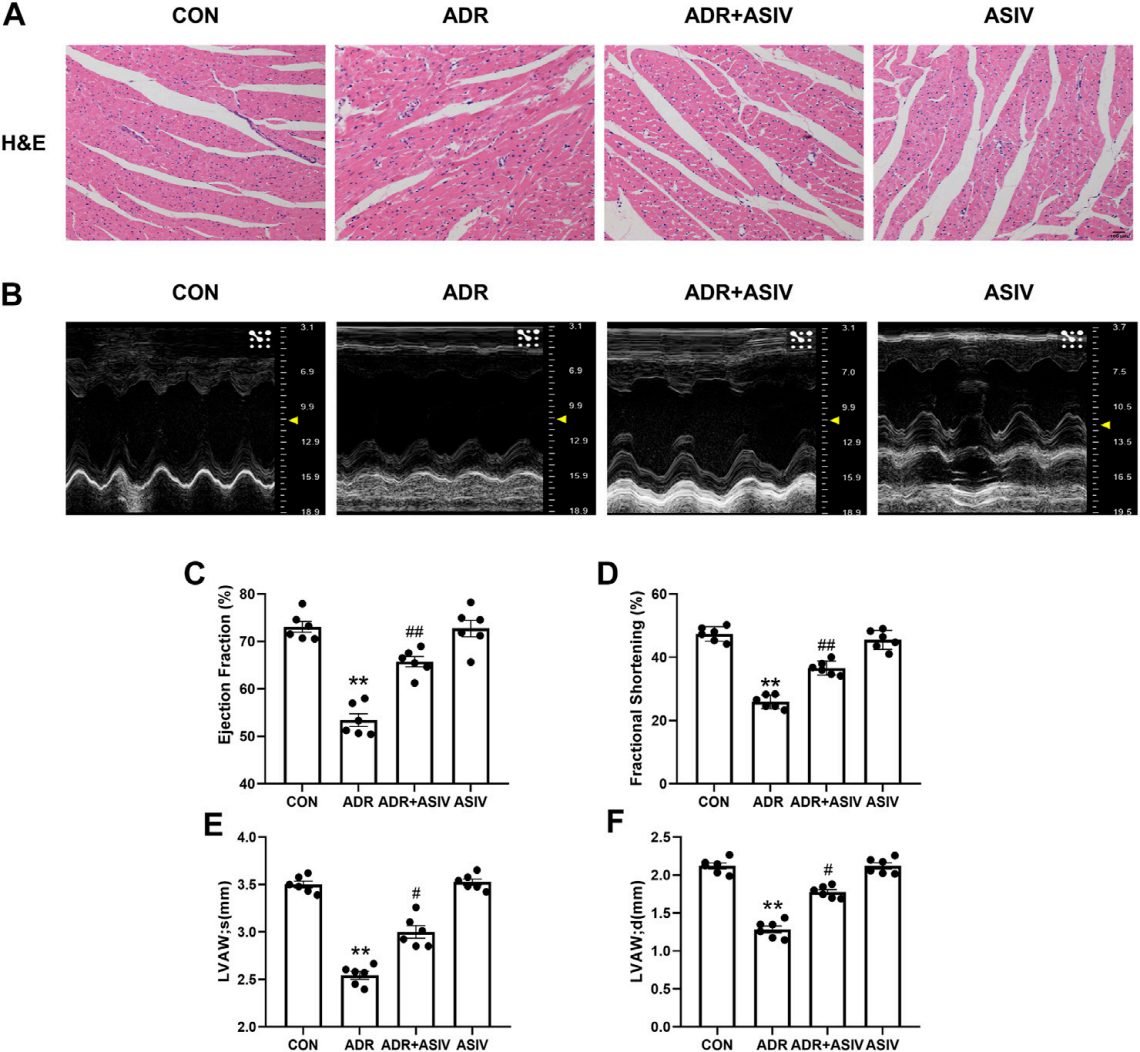
ASIV alleviates the ADR-induced impairment of cardiac function in rats. **(A)** Histological analysis of four heart sections (scale bar, 50 µm), **(B)** Representative ultrasound images of rats in different groups, **(C)** EF%, **(D)** FS%, **(E)** systolic thickness of the anterior wall of the left ventricle, **(F)** diastolic thickness of the anterior wall of the left ventricle of the four groups. Control and ADR groups, ADR + ASIV group and ASIV group. ADR, adriamycin; ASIV, astragaloside IV; FS, fractional shortening; EF, ejection fraction; LVAW; s, Systolic thickness of the anterior wall of the left ventricle; LVASW; d, diastolic thickness of the anterior wall of the left ventricle. *n* = 6. ^##^ *p* < 0.01 vs. the ADR group; **p < 0.01 vs. the control group.

To examine the effect of ASIV on ADR-induced myocardial structural changes, we observed HE-stained sections of the myocardium. Figure 1A shows that after the injection of ADR, significant morphological damage occurred in the myocardial tissues of the rats, including disordered cell arrangement, swelling and rupture of the cell nucleus. However, the administration of ASIV significantly preserved the myocardial structure.

Echocardiographic analysis (Figure 1B) showed that compared with the control rats, the ADR-treated rats exhibited significantly reduced left ventricular ejection fraction (Figure 1C) and fractional shortening (Figure 1D). However, these parameters were significantly restored in the ADR-treated rats administered ASIV. We found that the anterior wall of the left ventricle in the ADR group was significantly thinned in both the systolic (Figure 1E) and diastolic (Figure 1F) stages, while the ADR + ASIV group showed significant recovery.

### ASIV Reduces ADR-induced Oxidative Stress in the Heart

**FIGURE 2:**
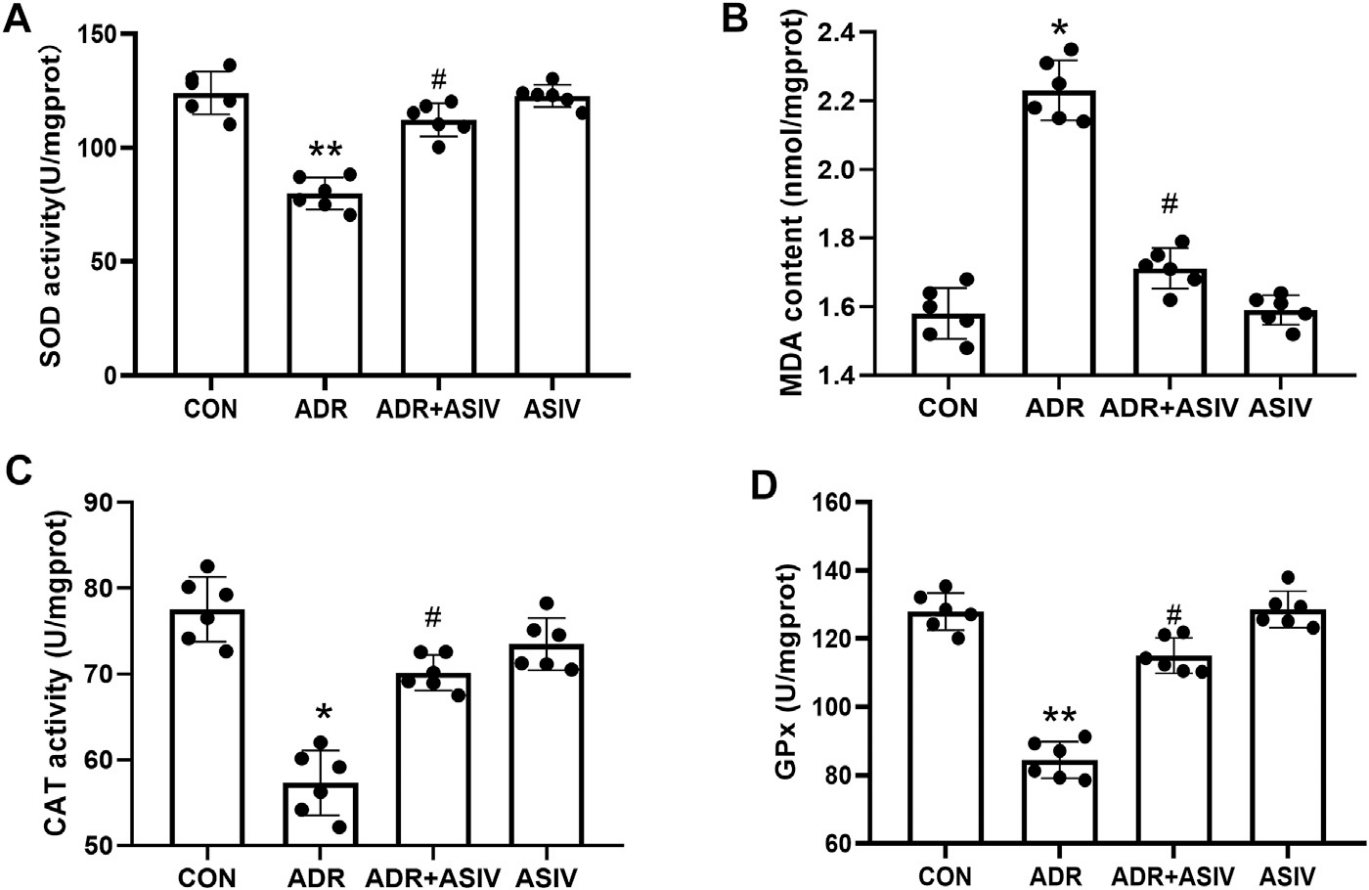
Effects of ASIV on myocardial oxidative stress markers in ADR-treated rats. **(A)** SOD activity, **(B)** MDA content, **(C)** CAT activity and **(D)** GPx activity in myocardial tissue. *n* = 6, *p < 0.05, **p < 0.01 vs. the control group; #*p* < 0.05 vs. the ADR group.

As shown in Figure 2B, the MDA content in the ADR group was significantly higher than that in the CON group, while the MDA content in the ASIV + ADR group was significantly lower than that in the ADR group. Figures 2A,C,D show the changes in the SOD, CAT and GSH-PX activities, respectively. The ADR group exhibited significantly lower activities than the CON group, and the ASIV + ADR group exhibited significantly higher activities than the ADR group. These results show that ASIV can inhibit the oxidative stress induced by adriamycin.

### ASIV Inhibits ADR-induced Cardiomyocyte Apoptosis

**FIGURE 3:**
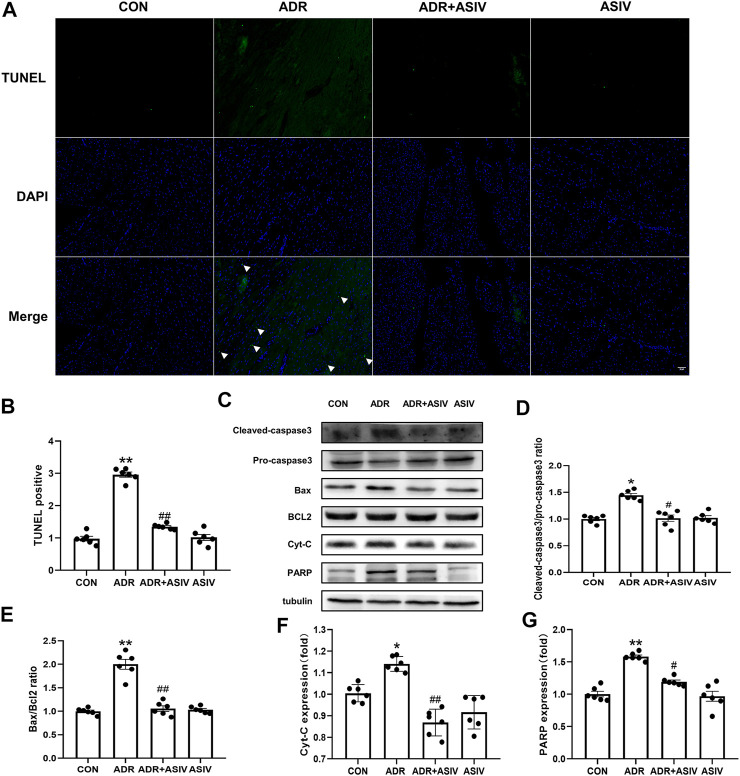
ASIV alleviates the ADR-induced apoptosis of rat cardiomyocytes. **(A)** The results of TUNEL staining of sections (scale bar, 100 µm), **(B)** Statistical analysis of the TUNEL-positive cells in the sections, **(C)** Western blotting results of cleaved-caspase3, pro-caspase3, Bax, BCL2, Cyt-C and PARP in myocardial tissue, **(D)** The ratio of cleaved-caspase-3 to pro caspase-3, **(E)** The ratio of Bax to BCL2, **(F)** Cyt-C protein expression level, **(G)** The protein expression level of PARP. *n* = 6. ^##^ *p* < 0.01, ^#^ *p* < 0.05 vs. the ADR group; **p < 0.01, *p < 0.05 vs. the control group.

TUNEL staining (Figure 3A) was used to observe the effect of ADR on cardiomyocyte apoptosis. The results showed that the TUNEL positivity (Figure 3B) of the ADR group was increased compared with that of the control group, and the index decreased after the administration of ASIV. Western blotting analysis of the cleaved-caspase3/pro-caspase3 (Figure 3D) and Bax/BCL-2 (Figure 3E) ratios also showed the same result; the ADR group exhibited significantly higher ratios than the control group, and the ratios were decreased after the administration of ASIV. Cyt-C (Figure 3F) and PARP (Figure 3G) are also markers of apoptosis. Cyt-C and PARP expression was significantly increased after ADR treatment, while Cyt-C and PARP expression was decreased after ASIV treatment.

### ASIV Reduces ADR-induced Autophagy

**FIGURE 4:**
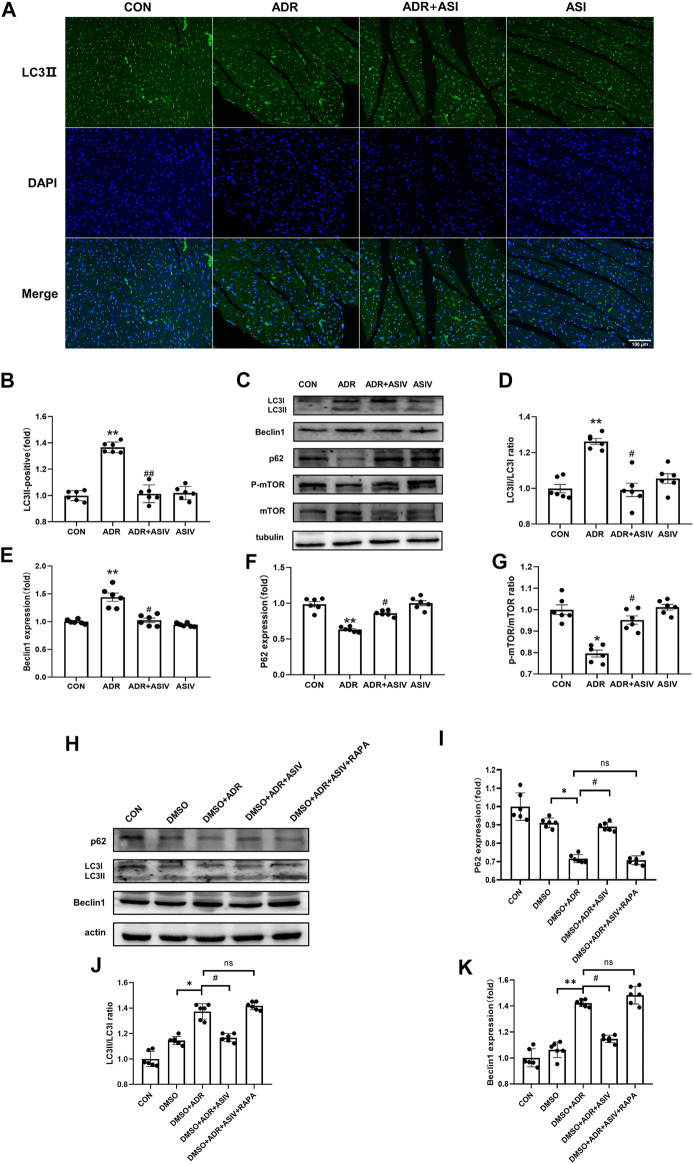
ASIV reduces ADR-induced autophagy. **(A)** Representative immunofluorescence images of LC3II staining (scale bar, 100 µm), **(B)** Semiquantitative analysis of LC3 II expression, **(C)** Representative Western blot ting images of the LC3, Beclin1, p62, *p*-mTOR and mTOR levels, **(D)** LC3II/LC3I ratio, **(E)** Beclin1 protein expression level, **(F)** p62 protein expression level, **(G)** *p*-mTOR/mTOR ratio, **(H)** Representative Western blotting images of LC3, Beclin1, and p62. **(I)** p62 protein expression level. **(J)** LC3II/LC3I ratio, **(K)** Beclin1 protein expression level. *n* = 6. ^##^ *p* < 0.01, ^#^ *p* < 0.05 vs. the ADR group; **p < 0.01, *p < 0.05 vs. the control group; ns, not significant.

We observed the effect of ASIV in regulating autophagy in ADR treated rats by detecting the levels of autophagy-related proteins. The immunofluorescence results (Figures 4A,B) showed that the contents of LC3II in the myocardial cells of the ADR group were higher than those of the other groups, while the contents of LC3II in the myocardial cells of the ADR + ASIV group were significantly decreased. The Western blotting results (Figure 4D) also showed that the ratios of LC3II/LC3I in the hearts of the ADR group were increased, while those in the ASIV + ADR group were decreased. The expression levels of Beclin-1 (Figure 4E) in the hearts of the ADR group were higher than those of the control group, while the expression levels of Beclin-1in the hearts of the ASIV + ADR group were decreased. The expression of p62 (Figure 4F) in the ADR group was decreased, and ASIV restored the expression of p62. These results proved that ADR increased the autophagic flux in rat cardiomyocytes and that ASIV reversed the abnormal increase in autophagy induced by ADR. mTOR is an important regulator related to protein synthesis and cell survival, and it is an important negative regulator of autophagy. This study examined the level of mTOR. The level of phosphorylated mTOR in the ADR group was lower than that in the control group, and the level of phosphorylated mTOR in the ADR + ASIV group was significantly recovered (Figure 4G).

We also used rapamycin to verify the effects of astragaloside IV *in vitro*. The Western blotting results showed that the ratios of LC3II/LC3I (Figure 4J) and the expression levels of Beclin-1 (Figure 4K) in the hearts of the ADR + DMSO group were higher than those in the hearts of the control group, while the ratios of LC3II/LC3I and the expression levels of Beclin-1 in the hearts of the ASIV + ADR + DMSO group were lower than those in the hearts of the ADR + DMSO group. The expression of P62 (Figure 4I) in the ADR + DMSO group was decreased, and ASIV restored the expression of p62. However, compared with the ADR + ASIV + DMSO group, the DMSO + ADR + ASIV + RAPA group exhibited significantly increased LC3II/LC3I ratios and Beclin-1 expression levels, and the expression level of p62 was significantly reduced. These results suggest that ASIV regulates ADR-induced cardiac autophagy by activating mTOR.

### Alteration in the Expression of Proteins Related to Biological Oxidative Protection in ADR-treated Rats by ASIV

**FIGURE 5:**
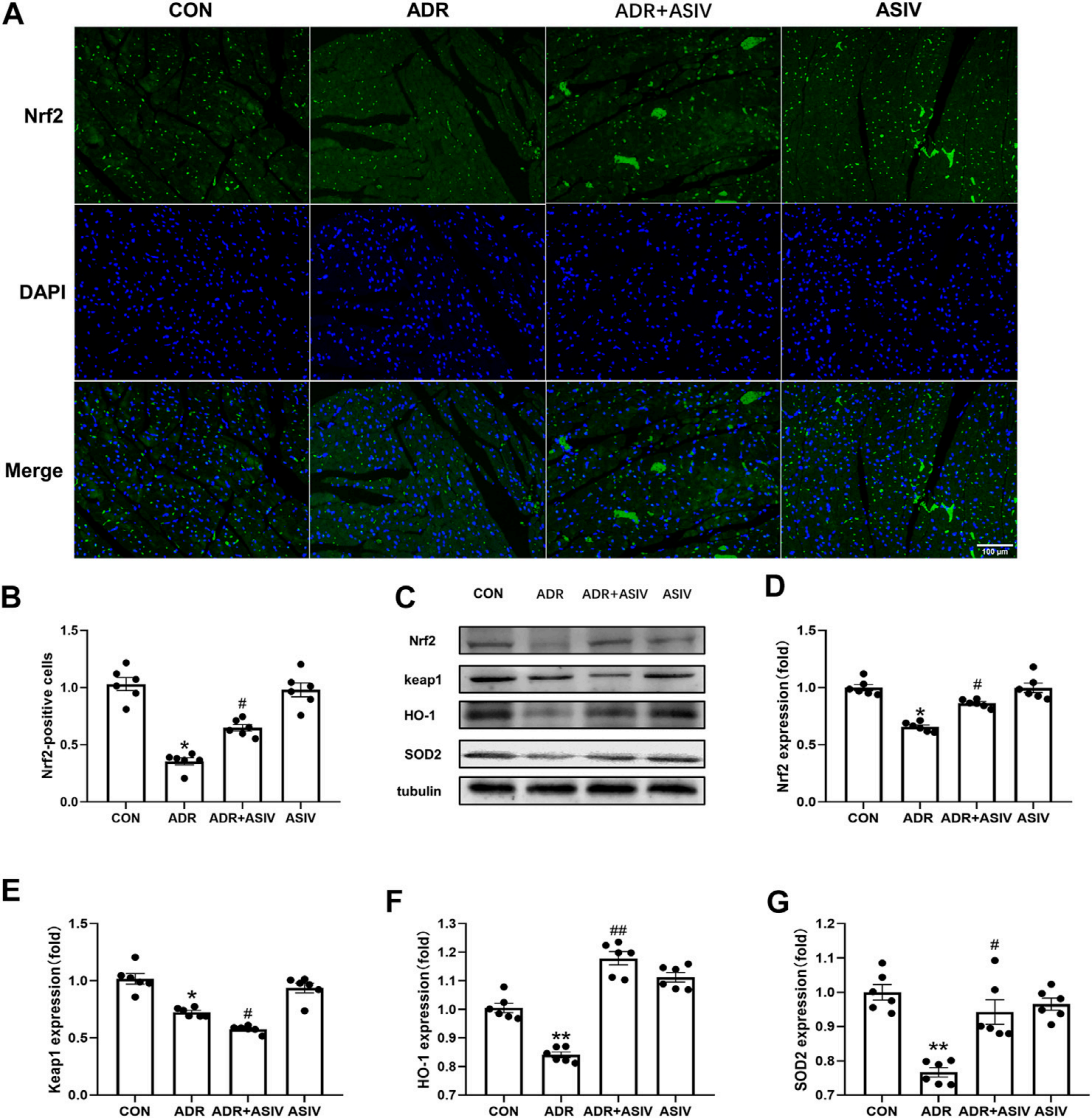
Alteration in the expression of proteins related to biological oxidative protection in ADR-treated rats by ASIV. **(A)** Representative immunofluorescent images of Nrf2 staining (scale bar, 100 µm), **(B)** Semiquantitative analysis of Nrf2 expression, **(C)** Western blotting results of Nrf2, Keap1, HO-1 and SOD2 in myocardial tissue, **(D)** Nrf2 protein expression level, **(E)** Keap1 protein expression level, **(F)** HO-1 protein expression level, **(G)** SOD2 protein expression level. *n* = 6. ^##^ *p* < 0.01, ^#^ *p* < 0.05 vs. the ADR group; **p < 0.01, *p < 0.05 vs. the control group.

Figure 5 shows that ADR downregulated the expression of Nrf2. However, after treatment with ASIV, Nrf2 expression was significantly increased (Figures 5A,B,D); Keap1 expression (Figure 5E) was significantly decreased in the ADR group, and it was further decreased in the ASIV + ADR group. HO-1 (Figure 5F) and SOD2 (Figure 5G) expression was significantly decreased after ADR treatment but increased after ASIV treatment.

**FIGURE 6:**
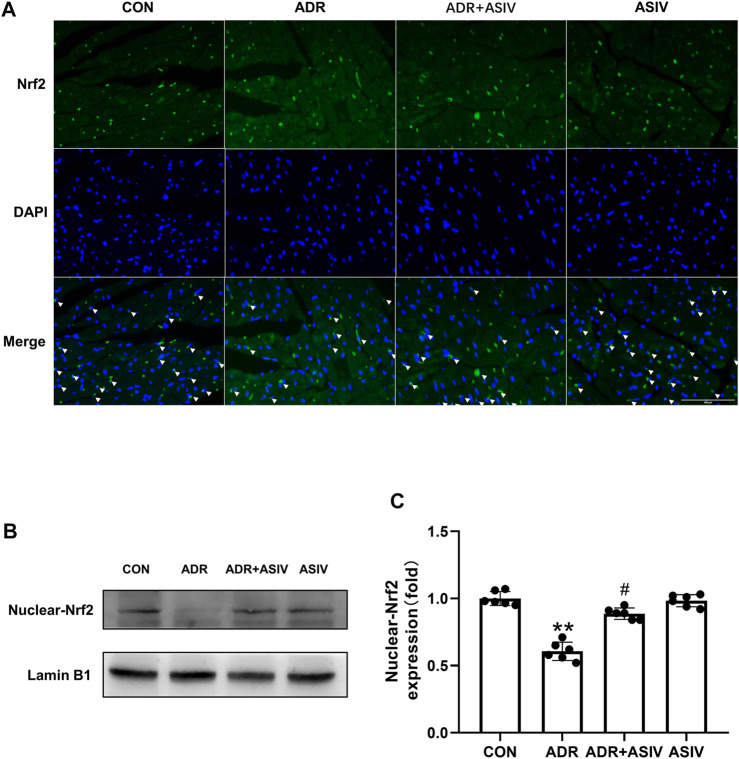
The effect of ASIV on Nrf2 in the nucleus of ADR-treated rats. **(A)** Immunofluorescence was performed to observe the nuclear localization of Nrf2 (scale bar, 100 µm), **(B)** Western blotting results of nuclear Nrf2 in myocardial tissue, **(C)** Nuclear Nrf2 protein expression level. *n* = 6. #*p* < 0.05 vs. the ADR group; **p < 0.01 vs. the control group.

Immunofluorescence was used to detect the nuclear translocation of Nrf2 (Figure 6A). The nuclear accumulation of Nrf2 in the ADR + ASIV group was significantly decreased, and the nuclear accumulation of Nrf2 in the ASIV treatment group was significantly increased. The ASIV group maintained the nuclear translocation of Nrf2. Changes in the nuclear accumulation of Nrf2 in cardiomyocytes were detected by Western blotting (Figure 6B). The results revealed that the contents of Nrf2 in the nuclei of the cardiomyocytes in the ADR group were significantly lower than those in the control group, and ASIV increased the contents of Nrf2 in the nuclei of the cardiomyocytes in the ADR group (Figure 6C). These results show that ASIV can reduce the oxidative stress caused by ADR by up-regulating Nrf2 expression.

### ASIV Regulates the PI3K/Akt Pathway in the Myocardium of ADR-treated Rats

**FIGURE 7:**
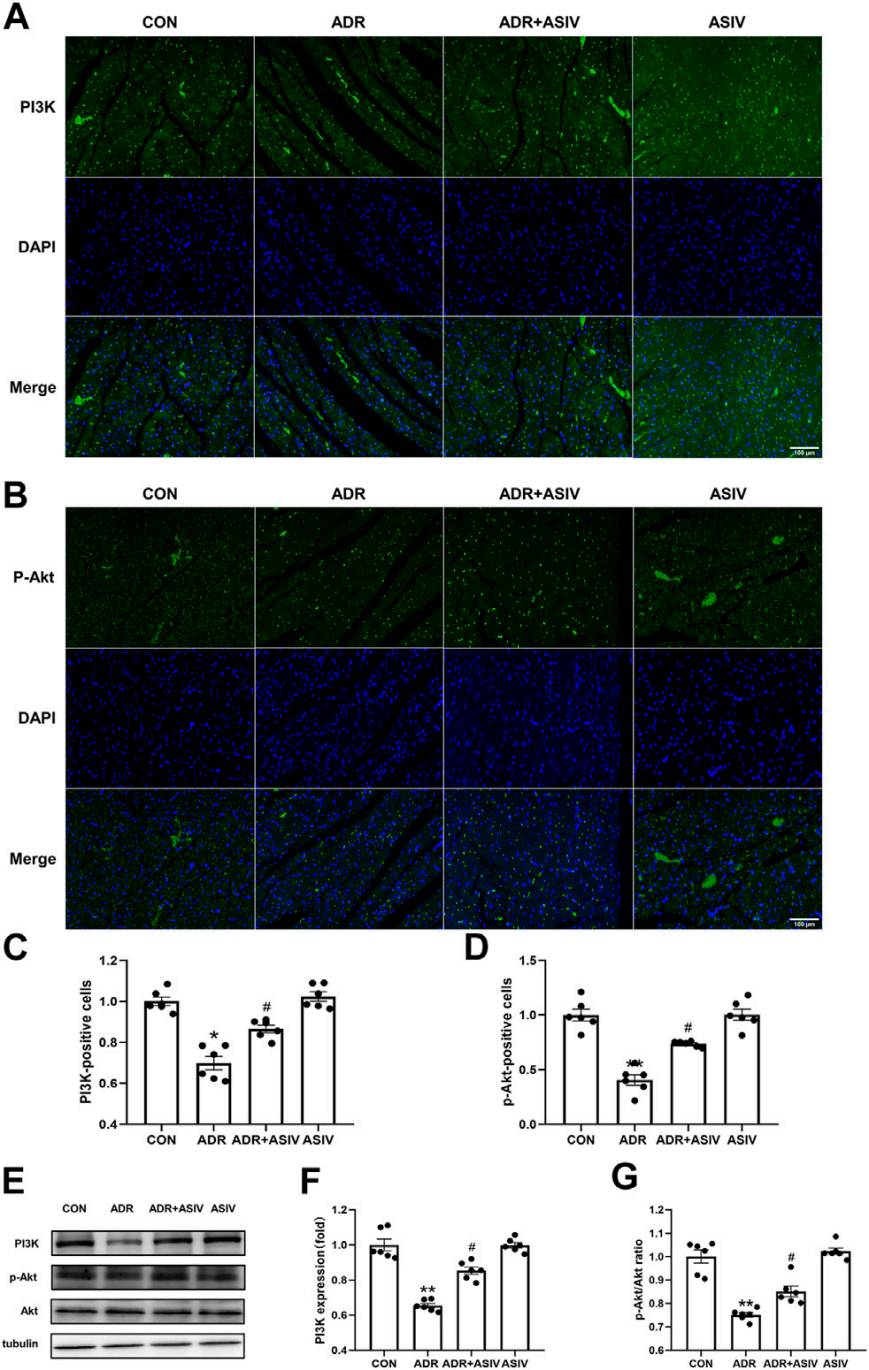
ASIV regulates the PI3K/Akt pathway in the myocardium of ADR -treated rats. **(A)** Representative immunofluorescence images of PI3K staining (scale bar, 100 µm), **(B)** Representative immunofluorescence images of *p*-Akt staining (scale bar, 100 µm), **(C)** Semiquantitative analysis of PI3K expression, **(D)** Semiquantitative analysis of *p*-Akt expression, **(E)** Western blotting results of PI3K, *p*-Akt and Akt in myocardial tissue, **(F)** PI3K protein expression level, **(G)** *p*-Akt/Akt ratio. *n* = 6. ^#^ *p* < 0.05 vs. the ADR group; **p < 0.01, *p < 0.05 vs. the control group.

The PI3K/Akt pathway can promote proliferation, cell survival, growth and metabolism in response to extracellular signals (Hoxhaj and Manning, 2020). The immunoblotting and immunofluorescence results showed that the expression of PI3K (Figures 7A,C,F) in the ADR group did not reach the level in the control group, but it recovered after ASIV administration. Then, we examined the downstream protein of PI3K, Akt. Consistent with the PI3K results, the *p*-Akt levels (Figures 7B,D) and *p*-Akt/Akt ratios (Figure 7G) of the ADR group were lower than those of the control group, and those of the ADR + ASIV group were significantly higher than those of the ADR group.

**FIGURE 8:**
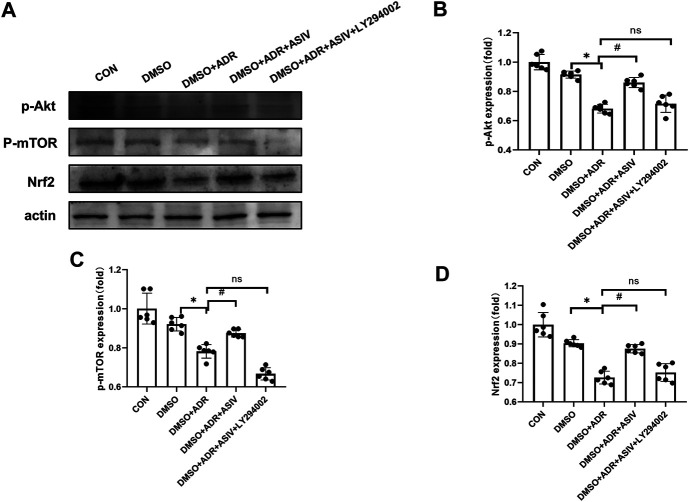
Inhibitors revealed that ASIV modulate the PI3K/Akt pathway in the myocardium ADR-treated cells. **(A)** Western blotting results of *p*-Akt, *p*-mTOR, and Nrf2 in cardiomyocytes after treatment with of inhibitors, **(B)** *p*-Akt protein expression level aftertreatment with inhibitors, **(C)** *p*-mTOR protein expression level after treatment with inhibitors, **(D)** Nrf2 protein expression level after treatment with inhibitors. *n* = 6. ^#^ *p* < 0.05 vs. the DMSO + ADR group; *p < 0.05 vs. the DMSO group; ns, not significant.

To further elucidate the role of the PI3K/Akt signaling pathway in the protective effect of ASIV on ADR-induced cardiotoxicity, we used LY294002 to inhibit the PI3K protein. Consistent with the results of the DMSO + ADR group, the activation of *p*-Akt (Figure 8B), *p*-mTOR (Figure 8C) and Nrf2 (Figure 8D) was reduced in the DMSO + ADR + ASIV + LY294002 group. This finding indicates that ADR inhibits mTOR and Nrf2 through the PI3K/Akt pathway, leading to increased oxidative stress and autophagy. ASIV can reduce the cardiotoxicity caused by adriamycin by regulating the PI3K/Akt pathway.

## Discussion

Our study investigated the effect of ASIV on ADR-induced myocardial injury in rats. There are several important findings of this research. 1. Decreased expression of p62 may be an important underlying mechanism of the cardiac oxidative stress caused by ADR. 2. ASIV reduces the level of cardiac oxidative stress in ADR-treated rats by inhibiting autophagy. 3. ASIV exert its inhibitory effect by activating the PI3K/Akt/mTOR pathway.

Autophagy is a mechanism by which cells respond to various internal and external nutrients, and it is an important cellular process; however, abnormal autophagy increases the prevalence of many diseases, including cardiovascular diseases (Sozen et al., 2020). Research suggests that adriamycin can increase cardiac autophagy levels to induce myocardial damage (Luo et al., 2018). mTOR is an important regulator that is related to protein synthesis and cell survival, and it is an important negative regulator of autophagy (Gu et al., 2020). The mTOR inhibitor rapamycin can induce autophagy by inactivating mTOR. By using rapamycin, we verified the discovery that adriamycin can cause heart damage by enhancing autophagy. Previous studies (Zhang et al., 2020a) have shown that adriamycin can enhance autophagy through PI3K/Akt/mTOR signaling. The protein level of PI3K in the heart is also related to HF. A decrease in the PI3Kα lipid kinase activity in the heart can lead to an increase in cardiac HF sensitivity. In animal models of late-stage HF and PI3Kα deficiency, reduced levels of p110α reduce the production of PIP3. Insufficient inhibition of gelsolin activity by PIP3 leads to excessive destruction of the cytoskeleton and damage the structural integrity of cardiomyocytes (Patel et al., 2018). Similar to our results, the rats injected with adriamycin showed obvious autophagic abnormalities; in particular, the expression of Beclin-1 and LC3II in the ADR group was increased, while the expression of p62 was decreased. The adriamycin treatment group exhibited significantly damaged heart function, and these results indicate that excessive autophagy is the causative factor of adriamycin-induced heart failure. In this experiment, ASIV ameliorated heart failure in the ADR-treated rats and reduced the level of cardiac autophagy. Our view is that ASIV stimulates PI3K and Akt to activate the downstream protein mTOR, thereby reducing the level of autophagy. This shows that ASIV can decrease the adriamycin-induced damage that causes heart failure by regulating autophagy. In addition to inducing excessive autophagy, adriamycin can also lead to defects in the formation of autophagic lysosomes. Studies have shown that adriamycin can increase the number of autophagosomes (Xu et al., 2013) and decrease the autophagic flux (Ma et al., 2017) by inhibiting cardiac autophagosome degradation. In this experiment, ASIV reduced the cardiac injury caused by adriamycin by regulating autophagy. Whether ASIV plays a role in the formation of autophagic lysosomes needs further experimental study.

The apoptosis of heart cells caused by adriamycin toxicity is inseparably related to heart failure (Wu et al., 2020), because heart cells nearly stop dividing after a period of time (Nguyen et al., 2020), and apoptosis promotes heart failure. In rats with ADR-induced heart failure, ADR induces an increase in the expression of proapoptotic cytokines, which is shown by the significant increase in the ratio of Bax/BCL-2. There is evidence (Ren et al., 2020) that PI3K/Akt signaling plays an important role in mediating cell apoptosis. The PI3K/Akt pathway can regulate the activity of BCL2 family members, which are regulators of the mitochondrial apoptosis pathway (Wang et al., 2020). Our results indicate that ASIV activates the PI3K/Akt pathway and significantly prevents adriamycin-induced apoptosis. There are two main mechanisms of apoptosis, internal and external (Luo et al., 2019). Activation of mitochondrial apoptosis leads to the intrinsic apoptotic signal transduction pathway. The activation of cytochrome c, apoptosis-inducing factor and caspases mediates the regulation of apoptosis by mitochondria. These factors are all regulated by the BCL2 protein family (Shan et al., 2020). In our experimental results, ASIV reduced the ADR-induced increases in the ratio of Bax/BCL-2 and the number of TUNEL positive cells, effectively inhibiting cardiomyocyte apoptosis.

It is generally believed that low levels of ROS promote intracellular signal transduction by activating autophagy, thereby promoting cell survival (Nishida et al., 2008). The activation of autophagy can protect the myocardium from apoptosis, and the autophagic flux during heart failure is an adaptive response that can protect cells from hemodynamic pressure (Zou and Xie, 2013). As the pressure increases, the activity of autophagy is insufficient to break down defective organelles and longevity proteins, leading to apoptosis (Nishida et al., 2008). The redox system may be the link between autophagy and apoptosis. We found that autophagy and apoptosis were simultaneously increased under the condition of high ROS levels induced by adriamycin. Astragaloside IV can inhibit the oxidative stress caused by high levels of ROS while reducing autophagy and apoptosis. Our findings indicate that the Nrf2 pathway may regulate the relationship between adriamycin-induced cardiomyocyte apoptosis and autophagy.

As an antioxidant, ASIV can activate the Nrf2 signaling pathway and inhibit ROS production (Nie et al., 2019). Upstream of Nrf2, PI3K/Akt is also an important pathway that regulates the cellular defense system to prevent oxidative damage, which is related to Nrf2 activation and HO-1 induction (Peng et al., 2020). Studies have found that blocking the activity of Akt by inhibitors can reduce the protein levels of Nrf2 and HO-1 (Kim et al., 2010). Experiments show that ASIV can significantly increase the phosphorylation of Akt and the expression of Nrf2 and HO-1 in ADR-treated rats, indicating that ASIV can activate the Akt/Nrf2/HO-1 signaling pathway. Oxidative stress is considered to be a key factor that affects the incidence of heart disease (Zhang et al., 2020c). Studies have shown that the cardiotoxicity caused by ADR is mainly related to the oxidative stress caused by ROS (Khadka et al., 2018), which is a complex multifactor process. It has been shown in the literature that the oxidative stress caused by ROS can directly induce autophagy and apoptosis (Zhang et al., 2020a).

Keap1 is an antioxidant transcription factor and a reverse regulator of Nrf2 (Kourakis et al., 2020). Existing data verify that adriamycin reduces the protein expression of Nrf2, but the expression of keap1 does not change with that of Nrf2. p62 is the specific autophagy receptor of Keap1 (Ghosh et al., 2020). P62 binds to Keap1 and combines with other ubiquitinated protein polymers for degradation and cause cell apoptosis (Zheng et al., 2020). Under normal conditions, the Nrf2-Keap1-p62 loop is in a dynamic and stable state, which can maintain cellular redox homeostasis (Luo et al., 2020). The excessive ROS levels caused by adriamycin *in vivo* induce autophagy, which prompts p62 to bind to Keap1 for degradation and subsequently inactivates Nrf2, leading to oxidative stress. However, the relationship among Nrf2, Keap1 and p62 is not a simple upstream and downstream relationship, but a two-way regulatory function (Zhang et al., 2020b). As shown in Figure 5, by activating the PI3K/Akt/Nrf2 pathway, ASIV upregulated the level of HO-1 to inhibit oxidative stress, inhibited the excessive autophagy caused by adriamycin, and activated the positive feedback loop of p62-Keap1-Nrf2 to continue to activate Nrf2. In short, oxidative stress, apoptosis and autophagy can affect each other through the p62, Keap1 and Nrf2 loops.

**FIGURE 9:**
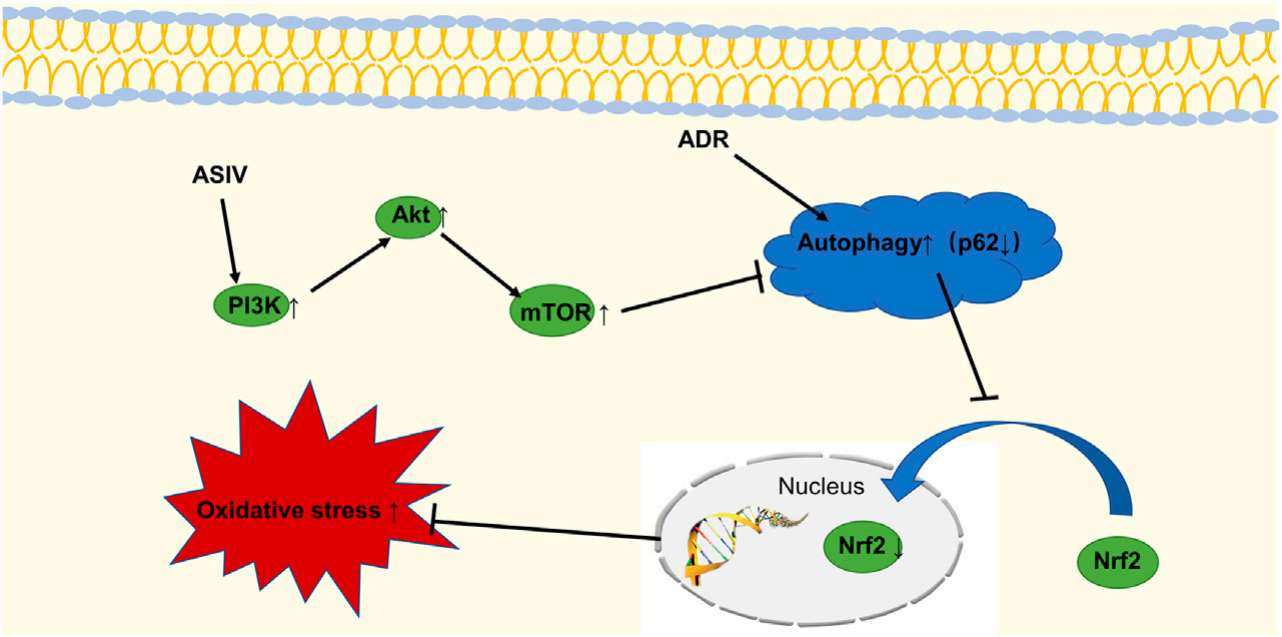
The schematic diagram shows that ASIV inhibits adriamycin-induced excessive autophagy by activating the PI3K/Akt pathway, upregulating Nrf2 expression, and inhibiting oxidative stress.

To verify our hypothesis, we blocked PI3K signaling with LY294002 to explore its possible mechanism. The results showed that ASIV reduced the adriamycin-induced increases in oxidative stress, autophagy and apoptosis by regulating the PI3K/Akt signaling pathway. We believe that as shown in Figure 9, astragaloside IV can protect against the heart damage caused by adriamycin by regulating the PI3K/Akt signaling pathway.

## Data Availability

The raw data supporting the conclusion of this article will be made available by the authors, without undue reservation.
